# An Electrical Disinfectant

**Published:** 1895-02

**Authors:** Albert E. Woolf

**Affiliations:** New York


### International Dental Journal.

Vol. XVI. February, 1895. No. 2.

### Original Communications.[1]

### AN ELECTRICAL DISINFECTANT.[2]

BY ALBERT E. WOOLF, NEW YORK.

Gentlemen,—I recall, just about this time, an epoch of my life when I was quite a boy. A number of us formed a little army and marched up to the Breevoort House, where Major Anderson was located, about the commencement of the war, and, making as much noise as we possibly could, called for Major Anderson. He came out and, after listening to an elaborate address, said, "Boys, I am a soldier, not a statesman, therefore you must excuse me for not making a speech to you. I thank you." Now, I may say, following the example of Major Anderson, if I am faulty in my delivery, I am an electrician and not an orator.

I am requested to appear before you to-night to give you an idea of what the electrical disinfectant is. I did not intend, before I came here, to go into details, neither did I intend to give the component parts of the disinfectant; but, coming in contact with a number of doctors who have been interested in the disinfectant and have experimented with it, I will trespass on your time a little and give you a slight history of the agent.

---

[1] The editor and publishers are not responsible for the views of authors of papers published in this department, nor for any claim to novelty, or otherwise, that may be made by them. No papers will be received for this department that have appeared in any other journal published in the country.

[2] Read before the Central Dental Association of Northern New Jersey, at the meeting November 19, 1894.

Many years ago, in experimenting with the storage battery, I found that after the plates were thoroughly charged there was present more or less peroxide of hydrogen. I well knew the value of peroxide of hydrogen, as I was the first to introduce it, about eighteen years ago, in the bleaching of ostrich feathers. Knowing its value both as a bleaching agent and also as an antiseptic, and finding it in my overcharged storage battery, I proceeded to attempt to make it with electricity. My experiments with different materials finally led to a chloride of sodium solution. The intermediate steps in which I arrived at results will not be sufficiently interesting to you for me to dwell on details. After utilizing the chloride solution and getting very good results from that, and knowing the quantity of chloride of sodium in sea-water, I experimented with it, and found still better results, so much so that its action on organic matter of vegetable origin was almost immediate and destructive. I began to experiment on germs, and experiments were also made in the Carnegie Laboratory and in the laboratory of the New York Board of Health, and the destructive power of this electrical disinfectant on germs was pronounced to be most effective, with the advantage of being non-poisonous. We then made some comparative tests with bichloride of mercury, carbolic acid, and sulphurous acid, and we found finally that bichloride of mercury, carbolic acid, and sulphurous acid were not germ-destroyers, particularly in the case of spore-germs. These agents rendered the spore-germs temporarily inactive, but they afterwards came to life, while the action of the electrical disinfectant is to decompose them entirely. We have brought the materials for some little experiments, which we will show you later on.

Now, the action of the electrical current on sea-water is this: We find in sea-water chloride of sodium, chloride of magnesium, and chloride of potassium, together with some iodides and bromides, as well as, I claim, almost every element known to nature. In the decomposition of chloride of sodium, for example, passing the electric current through the solution, we liberate at the positive pole chlorine and oxygen; at the negative the base sodium would be deposited and the hydrogen would escape. Oxygen and chlorine enter into the composition of the base and form hypochlorite. The same will be the case with the magnesium and potassium. Therefore hypochlorite of sodium, hypochlorite of potassium, and hypochlorite of magnesium are obtained from the decomposition of sea-water.

The experiments that have been made by bacteriologists show the destruction of germ-life by this electrical disinfectant to be rapid,—in fact, almost instantaneous,—while with the other so-called disinfectants it takes some time even to render the germs inactive. It has been a disputed question whether we have the formation of ozone in the decomposition of electrozone in presence of organic matter. The hypochlorites coming in contact with an organic body are split up; the chlorine, on account of its affinity for hydrogen, will seize on the hydrogen in the organic body, liberating therefrom one atom of oxygen. It will seize on the hydrogen of the moisture surrounding the organic body, liberating another atom of oxygen, and one atom is liberated from the hypochlorite. Thus there is liberated three atoms of nascent oxygen, which is ozone. Ozone is a powerful oxidizer, acknowledged to be as powerful as any ever discovered. I can only give you one illustration of its oxidizing action upon organic matter. It cannot be passed through a rubber tube without having an action on the rubber, while concentrated acids may be passed through the same rubber tube without having any action on it. Examining germs under the microscope, and bringing them in contact with the electrical disinfectant, we find motion ceases instantly. The reason of that is that the organic matter has been deprived of one of its elements. Then comes the oxidizing action, and under the microscope you will see the disintegration of the germ and the destruction of what has been heretofore germ-life. There is no disinfectant that I am aware of that will have that action on germs. The action of most disinfectants used heretofore is that of coagulation, thereby rendering the germs inert, but allowing them to remain in the same form as before, their shape not being changed; but if you take an elongated germ and treat it with the electrical disinfectant, it becomes in a short space of time contracted to almost a circular shape. All germs subjected to the action of the electrical disinfectant are contracted on account of the hydrogen being deprived of one element. While this has been accomplished with this disinfectant,—and the evidence that it has been accomplished is on the record,—then you will arrive at the conclusion that you can accomplish similar results in your particular specialties.

The first work that was done by the electrical disinfectant on a large scale was the treatment of the sewage of the town of Brewsters, New York. There the sewage, emptied on a marshy flat and being acted upon by the sun, caused a great amount of sickness; diphtheria, typhoid fever, typhoid malaria, scarlet fever, and kindred diseases were prevalent. The school-houses and public buildings were closed, and the peculiar situation and the mortality caused the authorities of New York to investigate in order to arrest the spread of disease. They found that the sewage of the town of Brewsters was contaminating the drinking-water of the city of New York by emptying into the Croton. The experiment which I had made before the Board of Health with this disinfectant induced them to consult me in the matter. I said I would construct a plant for treating the sewage of the town of Brewsters so as to render it perfectly innocuous. We constructed a plant there and treated the sewage. The effluent was afterwards tested by Dr. E. W. Martin, of the New York Board of Health; Dr. Dunham, of the Carnegie Laboratory; Drs. Berry and Beebe, and they found that the effluent was perfectly sterilized; in other words, the sewage of the town of Brewsters had been so treated that the effluent which ran into the drinking-water of the city of New York was perfectly free from contamination. A test was made of the sewage with iodide of-potassium paper, which was turned black, showing a surplus of gases over what were needed to sterilize it. We also treated the Croton water last year when it was in bad condition, showing nitrates and decomposed matter. We treated from seventy-five millions to eighty-five millions of gallons a day, and the water was proved to be, after treatment, free from foul and noxious gases.

Then the city of New York had deposited during the winter months a lot of garbage in a place called Riker's Island, which was bought by the city for this purpose. When the warm weather came fermentation set in, and there was a possibility of the formation of gases at that point sufficient to cause an upheaval. You couldn't go within four miles of Riker's Island when the wind blew from that direction without getting the odor of this fermenting garbage. We constructed a plant on the island for the purification of this mass of garbage, and to-day Riker's Island is free from odors or smells of any description, the decomposed matter deposited there is perfectly free from smell, and the large amount of sulphuretted hydrogen that was formed from the decomposing matter has all departed.

Thinking that a good many of you would be interested in this advance of science, I have brought here with me a box of garbage from Riker's Island, which I will pass around for your inspection. You will notice that it has a musty, mouldy smell, which does not appear in the presence of organic matter. The moment that musty, mouldy smell appears, you may know there is no organic matter present. Before we treated this garbage at Riker's Island you could put an egg down a few inches in any part of it and cook it by the heat that was generated from fermentation.

Now, if we can take putrid water—poisoned sewage—and render it innocuous; if we can take decomposed garbage and render it free from its offensive odors; if we can put that treated garbage under the microscope and show you that it is perfectly free from organic matter of all kinds; if we can take putrid meat in an advanced stage of decomposition and make it sweet; if we can instantly kill germs of all kinds wherever we find them, then we reach the point where you will understand that this disinfectant can be used in the treatment of the human tooth. There is not a particle of doubt in my mind that the destruction of teeth is due to the action of germ-life. As to what the form of germ-life is I plead ignorance; what the manner of attack on the teeth is I do not know; but that decay of the human teeth does emanate from germs there is not a particle of doubt. Let us see what, for instance, the action of toothache is. The enamel is attacked; the germ reaches the interior of the tooth, through its decay, and attacks the pulp. That it is a germ attacking the pulp of the tooth which causes toothache there is no doubt, because the application of specially prepared electrozone—I don't care how severe or intense the toothache is—will cure it. The moment it reaches through the hollow of the tooth to where the germ is, the pain ceases, which proves that the disinfectant has attacked the germs and they have ceased to irritate the pulp. That is my theory, and I think I am right.

By some little experiments that we will try later, you will see what the action of this disinfectant would be in cleansing teeth. If, before you fill a tooth, you wash it out with the disinfectant, also carefully washing the instruments to be used, and being sure that your hands are free from germs and animalculæ, you may fill the tooth successfully, without fear of a recurrence of the trouble. I have been informed that there are some here who have had experience with this disinfectant, and when I get through they will relate what their experience has been. Of all the disinfectants that you have been called upon in your practice to use, I do not know of any that you would put in the mouth of a patient without the feeling, every time you use it, that it will have some injurious effect and is objectionable in some shape or form; but one peculiarity of the electrical disinfectant is that it is perfectly harmless; it is non-poisonous.

[The speaker here took a generous drink of his disinfectant.]

As much as that [referring to what was drunk] of any other disinfectant that you know of would be injurious to very many human beings if taken internally, but here is one that you need have no fear of putting into the mouths of your patients. There are many liquids that people get into the habit of drinking, but you can rest assured that your patients will never get a liking for liquor in this shape. Now, in using this electrozone in dentistry, f you have any patients that you are particularly fond of, I think if you recommend to them the washing of the mouth with this disinfectant, night and morning, you will find that it will kill all germs that reach the teeth and prevent their attacks; in other words, if a child will use this disinfectant for cleansing the mouth, it will prevent decaying of the teeth.

I think that we will find out eventually what this disinfectant is and the uses to which it may be put. It may be manufactured on a large scale for flushing the streets and sewers of large cities, and for similar purposes. It can be made for such purposes at a remarkably low figure, to be used in large quantities. At Riker's Island we used ninety-four thousand gallons every day. I have sent to Dr. Brown some which was specially prepared for dental uses, and I do not want those here to assume that the disinfectant manufactured on a large scale for sterilizing garbage and similar uses is the same as that prepared for medicinal purposes. It is a different material altogether.

In the absence of the opportunity of showing you the action of this disinfectant upon germs, I have here a little illustration of its value as a destroyer of organic matter. The scientists to-day claim that all germs have their origin in decayed vegetation. It is a well-known fact that aniline dyes have their origin in the chemical changes of vegetation. Vegetable matter subjected to nature's action in the earth is converted into carbon, from carbon we get coal-oil, and from coal oil we make the aniline dyes. As we cannot show you the germs, we may make a test with coloring matter, the coloring matter being organic matter of vegetable origin, as the germs are organic matter of vegetable origin.

[The speaker here filled three glass tubes with colored liquid, and added to them respectively carbolic acid, sulphurous acid, and bichloride of mercury].

You see the action of carbolic acid, sulphurous acid, and bichloride of mercury. (I will say that these chemicals that I am using here are not my own, but were kindly furnished by the Association.) We will now take but one drop of the electrical disinfectant. You notice a white cloud appearing in that glass. A little agitation will decompose the organic matter; it is not the agitation alone that did the work. I showed this test several times in New York, and at first it was disputed by some scientists; they held that it merely decolored the water. I told them I did not know whether it only decolored the water or bleached the water, but the organic matter in it was decomposed and gone. The scientists will tell you to-day that all germ-creation is of albuminous growth. If that be a fact, and it is not disputed, then by experimenting with the white of an egg, which is pure albumen, we may see what the action would be upon germ-life. We will make some experiments in that way, which will give you an idea of how we can act upon germs, always bearing in mind the fact that germs are very minute, and that ten thousand cholera germs, placed side by side, would go through the eye of a needle; so, from the amount of albumen we are using here, you can form some idea of the many millions of colonies of germs that we are attacking. I have in this glass some pieces of broken glass. The object of it is to cut the albumen so that instead of one large globule of albumen we have to act on many little particles. [The speaker here broke an ancient egg.] Your President will be kind enough to tell you the condition of this egg. I will now put into it a few drops of the disinfectant. Your President will tell you the condition of the egg now.

*President Moore.*—It is a little more like Coney Island now.

*Mr. Woolf.*—Here we have some albumen which represents many colonies of germs. You notice that when I put bichloride of mercury, carbolic acid, or sulphurous acid in there, there is a coagulation of the outside of the mass of albumen. No matter how finely divided the albumen is in the tube, if you place a small particle of it under the microscope you will detect the presence of pure albumen inside and a coagulated coating on the outside. These so-called disinfectants form a coagulated coating on the outside, leaving the inside untouched. In like manner is the action on spore germs; the germ is rendered temporarily inert, but upon the disintegration of the coagulated coating on the outside the germ comes back to life again. Here are four tubes; I place in them sulphurous acid, carbolic acid, bichloride of mercury, electrical disinfectant. Now you notice coagulation here and coagulation there. You notice gas-bubbles commencing to rise here, which indicate disintegration of the albumen. Here you notice the albumen in coagulated form, and the same with the other two so-called disinfectants. There is no disputing the fact that the action of the disinfectant on germ life would be the same. If you noticed closely, you observed that before decomposition the liquid was pure white. It is rapidly turning yellow; the albumen is splitting up and is becoming part of the liquid; in a little while there will be no albumen there. When I turn this tube upside down some part of the albumen will remain in suspension, turning into gases without settling.

If it is at all interesting to those present, I would like to state that electrozone has proved a cure for tuberculosis of the rectum,—an affection where the tubercular germs enter the rectum and cause incessant diarrhœa. I have found that after two or three injections of this disinfectant the tubercular germ is killed and the diarrhœa is cured.

I believe it has been a problem that has caused a deal of anxiety to the medical profession to effect a cure where the cornea of the eye has commenced to slough. It usually results in the removal of one eye in order to save the other. Dr. Keyser, of Philadelphia, has used the electrical disinfectant in cases of sloughing cornea that he has treated, and in every instance a rapid cure followed. The doctor used it in a diluted form.

Dr. Roberts, of New York, had a patient suffering with a carbuncle, which commenced to slough, and blood-poisoning set in. He treated it by applying the electrical disinfectant on a cloth to the carbuncle, and in forty-eight hours the fever had broken.

These are a few of several cases that have been reported to me in which the electrical disinfectant has been tried at my request. I have received two letters within the last three weeks from patients treated for typhoid fever. It is reported to me that one patient who was being treated for typhoid fever was at death's door; they administered internally one part of the disinfectant to thirty parts of water, giving liberal injections also, and the patient recovered by reason of this treatment. In cases of diphtheria treated at North Brothers Island, New York, they have had better results from the use of the disinfectant than they have had from the use of the other medicines, and now the electrical disinfectant is used in almost all cases of diphtheria there.

I would also state that while the results of the treatment of cancer are at present, in my opinion, problematical, still, we have positively treated two, possibly three, cases of cancer, and one of them is reported as entirely cured.

I am sorry that Dr. Wallace is not here to-night, because he has had some experience in this connection with a case of cancer. There are many other cases in which the electrical disinfectant has been tried with excellent results.

While you have seen me drink this disinfectant, none of you are aware of its properties and the large volume of gases contained in it. To make the thing perfectly plain, I will decompose some with sulphuric acid. The action of the acid will be to split up the hypochlorites and liberate the gases. That sulphuric acid plays no part in the liberation of the fumes without the disinfectant I will show you by first placing some in water. I have here some ammonia to show the amount of chlorine liberated; I will put the fume of ammonia around it. First, I will show you that the ammonia in itself plays no perceptible part.

I will take some disinfectant now and I will split up the disinfectant with sulphuric acid; that will give you an idea of why it is so potent—the amount of gases it contains—to do the work which it is reputed to do.

Now, if we have here a non-poisonous disinfectant, one that can be used without fear of injury, that you can apply to an ulcer with assurance that the discharge will be stopped, that you can use in the mouth, or as an injection, without doing harm, we have what you have all been looking for. I say, if you have here a perfectly harmless material, you are justified in experimenting with it on the suffering, and I think you will find that after you have experimented with it you will be highly edified. As to its application in cases of eczema, I will say that, while we have failed in some cases, we have received enough encouragement, certainly, to warrant a continuation of experimentation in that direction.

I am told that there are some doctors here from Philadelphia. The head of the Home for Consumptives in that city, Dr. J. Solis Cohen, has tried the electrical disinfectant at the Home for Consumptives, and he reports that the "ward in which he used the electrozone is much sweeter than the other wards, although all would be considered sweet until the one in question is entered." He reports that among the patients where he has used the electrozone expectoration has diminished in a marked degree. He hopes, after further experimentation, to report some further good results. My personal opinion is that if, instead of dividing the disinfectant into atoms by means of an atomizer, it were vaporized finely, so that it would be more easily drawn into the ramifications of the tubes entering the lungs, there would be a good chance of beneficial results.

I have recited these few cases to you in order that you may benefit from my experience, and positively with no other object; for I feel that if I should in any manner be instrumental in easing pains and sufferings, then possibly I would have added my little modicum of plaster to the statue of science, which will not have been completed when time shall cease to be.

If any of you gentlemen have the inclination to ask questions, I am at your disposal to answer any that you may wish to put to me. I have a microscope here, and have brought some germs also, and we will put the germs under the microscope for your inspection and edification.

